# Dysregulation of the mTOR Pathway Mediates Impairment of Synaptic Plasticity in a Mouse Model of Alzheimer's Disease

**DOI:** 10.1371/journal.pone.0012845

**Published:** 2010-09-20

**Authors:** Tao Ma, Charles A. Hoeffer, Estibaliz Capetillo-Zarate, Fangmin Yu, Helen Wong, Michael T. Lin, Davide Tampellini, Eric Klann, Robert D. Blitzer, Gunnar K. Gouras

**Affiliations:** 1 Department of Neurology and Neuroscience, Weill Cornell Medical College, New York, New York, United States of America; 2 Center for Neural Science, New York University, New York, New York, United States of America; 3 Department of Pharmacology and Systems Therapeutics, Mount Sinai School of Medicine, New York, New York, United States of America; 4 Rockefeller University, New York, New York, United States of America; Brigham and Women's Hospital, Harvard Medical School, United States of America

## Abstract

**Background:**

The mammalian target of rapamycin (mTOR) is an evolutionarily conserved Ser/Thr protein kinase that plays a pivotal role in multiple fundamental biological processes, including synaptic plasticity. We explored the relationship between the mTOR pathway and β-amyloid (Aβ)-induced synaptic dysfunction, which is considered to be critical in the pathogenesis of Alzheimer's disease (AD).

**Methodology/Principal Findings:**

We provide evidence that inhibition of mTOR signaling correlates with impairment in synaptic plasticity in hippocampal slices from an AD mouse model and in wild-type slices exposed to exogenous Aβ1-42. Importantly, by up-regulating mTOR signaling, glycogen synthase kinase 3 (GSK3) inhibitors rescued LTP in the AD mouse model, and genetic deletion of FK506-binding protein 12 (FKBP12) prevented Aβ-induced impairment in long-term potentiation (LTP). In addition, confocal microscopy demonstrated co-localization of intraneuronal Aβ42 with mTOR.

**Conclusions/Significance:**

These data support the notion that the mTOR pathway modulates Aβ-related synaptic dysfunction in AD.

## Introduction

Increasing evidence supports the idea that in Alzheimer's disease (AD) functional impairment of synaptic plasticity develops before neurodegeneration. Extensive research has shown that soluble oligomers of β-amyloid peptide (Aβ), cleaved from the amyloid precursor protein (APP), are capable of inhibiting long-term potentiation (LTP) and causing learning and memory deficits [1], [2]. Formation of Aβ-derived plaques, a pathological hallmark of AD, develops after accumulation of soluble Aβ oligomers. These findings have focused attention on the early, pre-plaque stage of AD when synaptic plasticity is already impaired by Aβ [3], [4]. One of the central questions is how abnormal Aβ accumulation in the brain causes synaptic dysfunction and thus cognitive deficits. The molecular signaling mechanisms through which Aβ exerts its synapto-toxic effects remain poorly understood.

Mammalian target of rapamycin (mTOR), an evolutionarily conserved serine/threonine protein kinase, plays an essential role in the control of protein translation and cell growth by responding to multiple environmental cues including growth factors, nutrient state, and energy level, among others. Its importance in cellular and organismal homeostasis is reflected in the association of dysregulated mTOR signaling with common diseases such as cancer and diabetes [5]. More recently, mTOR has also been shown to be important for neurons. Next to its role in long-term synaptic plasticity, emerging evidence implicates mTOR in axon pathfinding and regeneration, dendrite arborization and spine morphology [6]. Control of protein translation by mTOR occurs via phosphorylation of at least two well-established downstream targets: p70 S6 kinase (p70S6K) and a repressor protein of the cap-binding eukaryotic initiation factor 4E (eIF4E) termed eIF4E-binding protein (4E-BP). A major upstream regulator of mTOR is tuberous sclerosis complex 2 (TSC2), which integrates signals from many other signaling molecules, including Akt (PKB) and glycogen synthase kinase 3 (GSK3) [5].

Increasing evidence has pointed to a link between mTOR and AD. First, mTOR is critical for long-lasting forms of synaptic plasticity and long-term memory (LTM) formation [7], which is impaired in mouse models of AD. The importance of mTOR in synaptic plasticity is in agreement with the central role of mTOR in controlling mRNA translation, since *de novo* protein synthesis is involved in these long-lasting forms of synaptic plasticity and LTM [8]. Second, inhibition of the mTOR pathway was shown to modulate aging, a well-established risk factor for AD [9], [10]. Third, autophagy, a pathway for organelle and protein turnover, has been implicated in the neurodegeneration of AD, and the well-characterized mTOR inhibitor, rapamycin, is known to induce autophagy [11]. Finally, mTOR signaling has been shown to be altered in AD models, although data appears to be conflicting. Down-regulation of mTOR signaling was reported in neuroblastoma cells treated with Aβ1-42 and in brains of APP/PS1 mutant transgenic mice [12]. In contrast, mTOR signaling was shown to be up-regulated in 7PA2 cells over-expressing mutant APP and in brains of another AD transgenic mouse, with rapamycin treatment reported as protective against behavioral decline [13], [14]. However, whether these signaling changes were related to alterations in synaptic plasticity was not explored in these studies.

In the current study we asked whether the mTOR signaling pathway is involved in the well-established Aβ-induced impairment of synaptic plasticity. We report that mTOR signaling is inhibited both in cultured neurons and hippocampal slices from AD transgenic mice and in wild-type (WT) hippocampal slices exposed to exogenous Aβ1-42, and that this mTOR dysregulation correlated with impairment in synaptic plasticity. Importantly, up-regulation of mTOR signaling by both pharmacological and genetic methods prevented Aβ-induced synaptic impairment, supporting the notion that dysregulation of the mTOR pathway is critical for the synaptic dysfunction that characterizes AD.

## Results

### Inhibition of mTOR signaling correlates with impairment of synaptic plasticity in an AD mouse model

**Figure 1:**
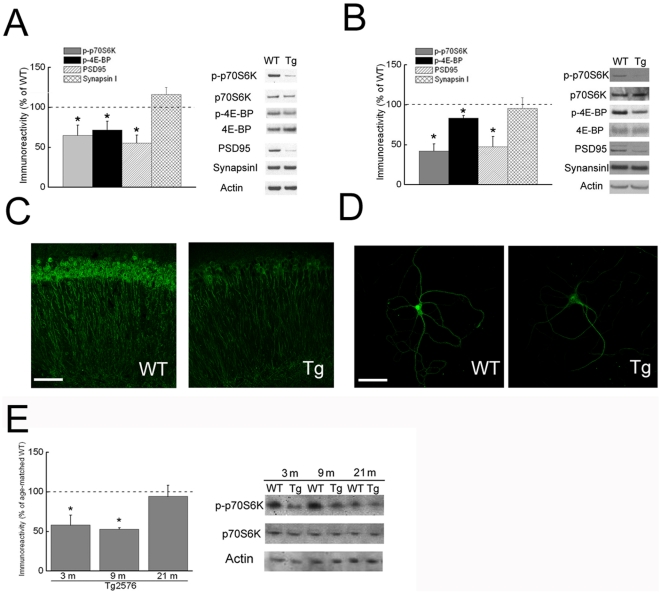
mTOR signaling is impaired in an AD mouse model. (A) Western blot of acute hippocampal slices from 3–4-month-old Tg2576 mice showed decreased levels of phospho-p70S6K (Thr389), phospho-4E-BP (Thr37/46) and PSD-95 compared to wild-type (WT) slices. n = 9. *p<0.05. (B) Western blot on cultured primary neurons at 12 DIV showed decreased levels of phospho-p70S6K, phospho-4E-BP and PSD-95 in Tg2576 compared WT neurons. n = 4. *p<0.05. (C and D) Immuno-fluorescence confocal microscopy for phospho-p70S6K in the CA1 region of hippocampal slices (C) and cultured primary neurons at 12 DIV (D) showed reduced immuno-staining in Tg2576 compared to WT mice. Representative images are shown from three experiments. Scale bar, 75 µm. (E) In contrast to 3 and 9 month-old mice, reduced phospho-p70S6K was no longer evident of brains from aged (21–month-old) Tg2576 compared to WT mice. n = 8 for 3-month-old; n = 4 for 9-month-old; n = 4 for 21–month-old. *p<0.05.

Increasing evidence points to a critical role for mTOR in synaptic plasticity, aging and autophagy, all of which have been linked to AD. We therefore examined whether the mTOR pathway is altered in the well-established Tg2576 AD transgenic mouse model. Tg2576 mice harbor the human APP transgene with the Swedish mutation and develop AD-like amyloidosis and memory deficits [15]. Western blotting was performed in acute hippocampal slices from 3–4 month-old Tg2576 mice, an age well before the development of Aβ plaques but at which time early physiological and functional deficits have been described [16], [17]. Compared to wild-type (WT) littermate slices, hippocampal slices from Tg2576 mice showed a significant decrease in the levels of p70S6K phosphorylated at threonine 389 (phospho-p70S6K), a site phosphorylated by mTOR and used as a readout for mTOR activity [18] (Figure 1A). In addition, levels of 4E-BP phosphorylated at threonine 46/47 (phospho-4E-BP) were reduced in hippocampal slices from Tg2576 compared to WT mice (Figure 1A). There was an analogous pattern of changes in levels of phosphorylated p70S6K and 4E-BP in Tg2576 compared to WT neurons in culture (Figure 1B). To further confirm reduced phosphorylation of p70S6K in Tg2576 compared to WT mice we performed immuno-fluorescence confocal microscopy. Consistent with the biochemical data, immuno-fluorescence of phospho-p70S6K was also reduced in both hippocampal slices (3–4-month-old) and primary neurons (12 DIV) of Tg2576 compared to WT mice (Figures 1C and 1D).

Cultured Tg2576 neurons were previously reported to have early Aβ-dependent reductions in PSD-95 but not in synapsin I [19], [20]. We now provide evidence that levels of PSD-95 are similarly reduced and levels of synapsin I unchanged in hippocampal slices from Tg2576 compared to WT mice (Figure 1A).

Since brains from advanced human AD were shown to have isolated increases in phospho-p70S6K, particularly in tangle-bearing neurons [21], we next examined phospho-p70S6K in Tg2576 transgenic mouse brains with aging. Of note, the reduced phospho-p70S6K observed in young 3–4 month-old Tg2576 compared to wild type mice was no longer evident in brain tissue from older (21 month-old) Tg2576 mice (Figure 1E).

**Figure 2:**
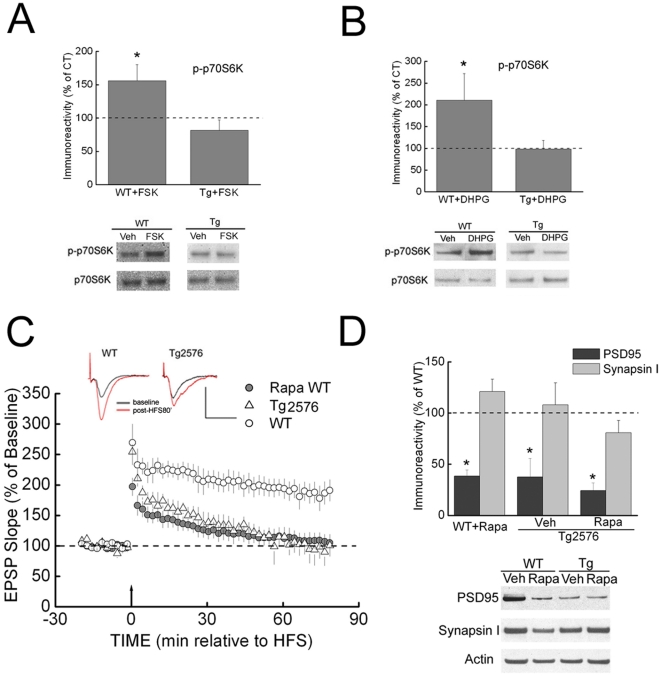
mTOR signaling dysfunction correlates with synaptic plasticity impairment in an AD mouse model. (A) Treatment of slices with forskolin (FSK, 50 µM, 60 min) induced phosphorylation of p70S6K in WT (n = 10) but not Tg2576 mice (n = 11). Values of densitometry from drug-treated slices are normalized to their vehicle control. *p<0.05. (B) Treatment of slices with 3,5 dihydroxyphenylglycine (DHPG, 50 µM, 60 min) induced phosphorylation of p70S6K in WT (n = 9) but not Tg2576 mice (n = 6). Values of densitometry from drug-treated slices are normalized to their vehicle control. *p<0.05. (C) High frequency stimulation (HFS) induced normal LTP in slices from 3–4-month-old WT mice (open circles), but only decremental LTP in Tg2576 mice (triangles). Pretreatment of slices with rapamycin (Rapa, 1 µM, 30 min) blocked LTP induced in WT mice (dark gray circles). n = 4 for WT and Tg; n = 6 for Rapa treated WT. Rapamycin was present throughout the recording. Scale bar, 1 mV/20 ms. The inset traces show superimposed sample EPSPs recorded during the baseline period (black) and 60 min after HFS (red). (D) Treatment of slices with Rapa (1 µM, 120 min) reduced levels of PSD-95 in WT but not Tg2576 mice. n = 5. *p<0.05.

Activation of the mTOR pathway is tightly associated with both LTP and long-term depression (LTD), two important forms of synaptic plasticity [7]. Therefore, we next treated slices either with the adenylyl cyclase activator forskolin (FSK) or the mGluR1/5 agonist 3,5-dihydroxyphenylglycine (DHPG), which are well-established to induce chemical LTP and LTD, respectively [22], [23]. Levels of phospho-p70S6K were significantly up-regulated in both FSK- and DHPG-treated slices from WT mice, but these responses were markedly blunted in slices from 3–4 month-old Tg2576 mice (Figures 2A and 2B). These data are consistent with impaired activation of mTOR signaling in hippocampal slices of Tg2576 mice.

Next we examined alterations in synaptic plasticity in Tg2576 mice by inducing LTP with high-frequency stimulation (HFS) at CA3-CA1 synapses of acute hippocampal slices. Of note, LTP was inhibited in slices from 3–4-month-old Tg2576 compared to WT mice (Figure 2C). In contrast, LTP was normal in slices from younger (2-month-old) Tg2576 mice (Figure S1A). Consistent with the absence of an LTP phenotype, Western blotting of 2-month-old Tg2576 slices also demonstrated no alterations in levels of phosphorylated p70S6K or PSD-95 at this age (Figure S1B). These data are consistent with prior work showing the onset of abnormalities in Tg2576 mice at 3–4 months of age [16], [17].

We next confirmed previous work showing that LTP in WT slices was rapamycin-sensitive [24] and noted that the decay of LTP was similar to that in Tg2576 hippocampal slices (Figure 2C). To further compare the effects of down-regulation of mTOR signaling by rapamycin to alterations seen in hippocampal slices from Tg2576 mice, expression of selective synaptic proteins was examined in the presence of rapamycin. In WT hippocampal slices treated with rapamycin, levels of PSD-95 but not synapsin I were significantly decreased (Figure 2D), similar to the changes seen in Tg2576 slices (Figure 1A). However, when Tg2576 slices were treated with rapamycin, there was no further decrease in the levels of PSD-95 (Figure 2C).

The HFS protocol used in our experiments is known to induce long-lasting, protein synthesis- and mTOR-dependent LTP [24]. In contrast, weaker HFS, which induces protein synthesis- and mTOR-independent early LTP, revealed no difference in LTP between Tg2576 and WT slices (Figure S2A). In addition, paired-pulse facilitation (PPF) experiments were performed to assess pre-synaptic plasticity [25], and no significant difference was observed between 3–4-month-old Tg2576 and WT mice (Figure S2B). Taken together, the data described above indicate that impaired up-regulation of mTOR signaling and impaired LTP both occur in hippocampal slices from 3–4 month old Tg2576 mice, whereas neither occurs at 2 months of age. Thus, impaired upregulation of mTOR signaling correlates with an impairment in LTP, supporting a link between the mTOR signaling pathway and AD-related synaptic dysfunction.

### Up-regulation of the mTOR pathway rescues Aβ-related inhibition of LTP

**Figure 3:**
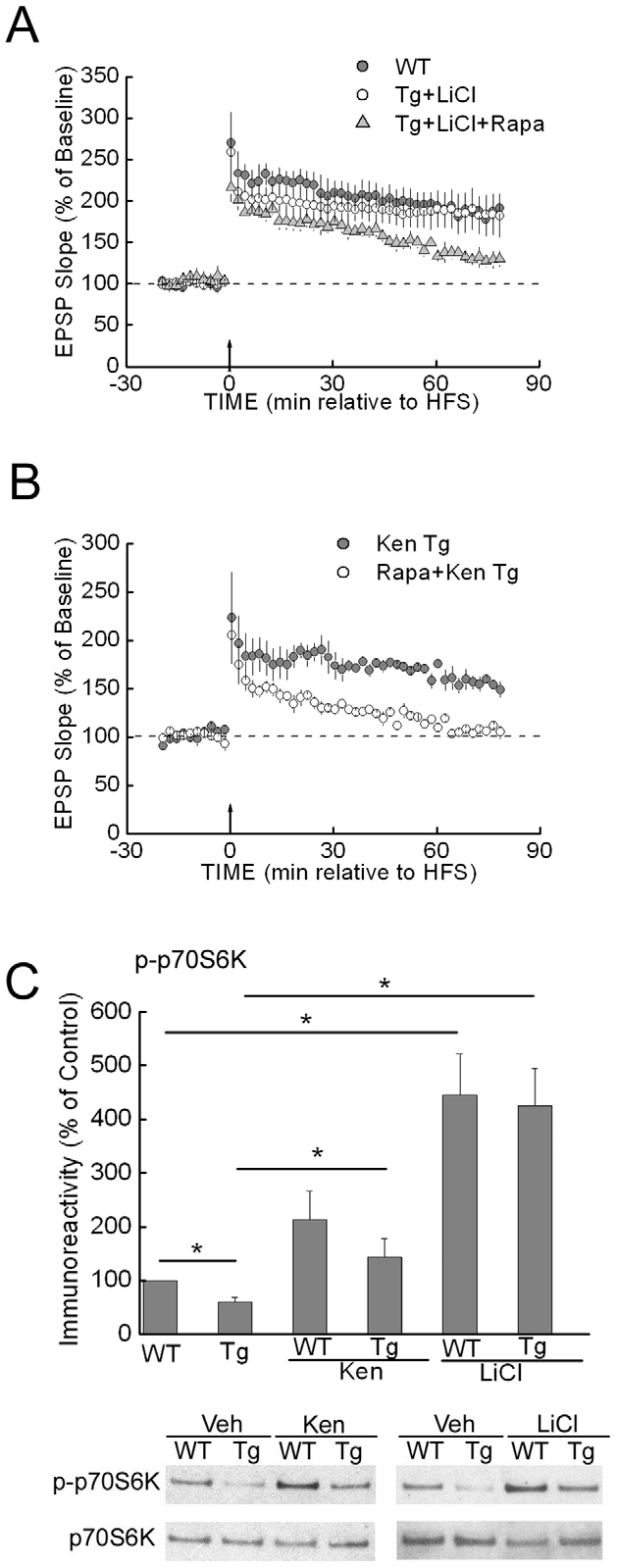
Up-regulation of the mTOR pathway via GSK3 inhibition rescues LTP impairment in Tg2576 mice. (A) Compared to the LTP induced in hippocampal slices of WT mice (filled circles, n = 4), high frequency stimulation (HFS) induced normal LTP in slices from Tg2576 mice treated with LiCl (10 mM, open circles, n = 5), which was inhibited by 30 min of rapamycin (Rapa; 1 µM) pretreatment (gray triangles, n = 4). LiCl or rapamycin was present throughout the recording. (B) Similarly, treatment of slices from Tg2576 mice with kenpaullone (Ken, 5 µM, dark gray circles) prevented the impaired HFS induced LTP seen in untreated Tg2576 slices; the protection in LTP induction in Tg2576 slices by kenpaullone was inhibited by 30 min of rapamycin (1 µM) pretreatment (open circles). n = 3. Kenpaullone or rapamycin was present throughout the recording. (C) The decreased levels of phospho-p70S6K in hippocampal slices from Tg2576 compared to WT (control) mice was prevented by treatment with either Ken (5 µM) or LiCl (10 mM) for 60 min. n = 8. *p<0.05.

Because down-regulation of mTOR signaling in Tg2576 mouse hippocampal slices correlated with impairment in synaptic plasticity, we next examined whether increasing mTOR signaling could protect against Aβ-related impairment in LTP. The major negative upstream regulator of mTOR is the TSC2 complex, which integrates multiple kinase inputs, including Akt and GSK3. It is known that GSK3 inhibits mTOR signaling by phosphorylating TSC2. Since TSC2 is positively regulated by GSK3, blocking GSK3 activity releases the inhibitory effect of TSC2 on mTOR and thereby increases mTOR activity [26], [27]. Moreover, inhibition of GSK3 activity has been shown to strengthen hippocampal LTP [28]. In the presence of structurally distinct GSK3 antagonists LiCl (10 mM) or kenpaullone (5 µM), HFS was now able to induce long-lasting LTP in slices from Tg2576 mice (Figures 3A and 3B), in comparison to only decremental LTP in Tg2576 slices in the absence of GSK3 antagonists (Figure 2C). To confirm that this potentiation of LTP in Tg2576 slices is dependent on mTOR signaling, we determined whether LiCl- or kenpaullone-augmented LTP was blocked by rapamycin. Slices were pretreated with rapamycin for 30 min, followed by application of LiCl or kenpaullone in the presence of rapamycin. Rapamycin treatment prevented enhancement of LTP by either LiCl or kenpaullone in Tg2576 hippocampal slices (Figures 3A and 3B). Furthermore, Western blotting demonstrated that levels of phosphorylated p70S6K were significantly elevated by treatment of Tg2576 hippocampal slices with either LiCl or kenpaullone (Figure 3C), providing support that up-regulation of mTOR signaling is involved in the rescued LTP in Tg2576 slices.

### Inhibition of mTOR signaling and LTP by Aβ is prevented in FKBP12 conditional knockout mice

**Figure 4:**
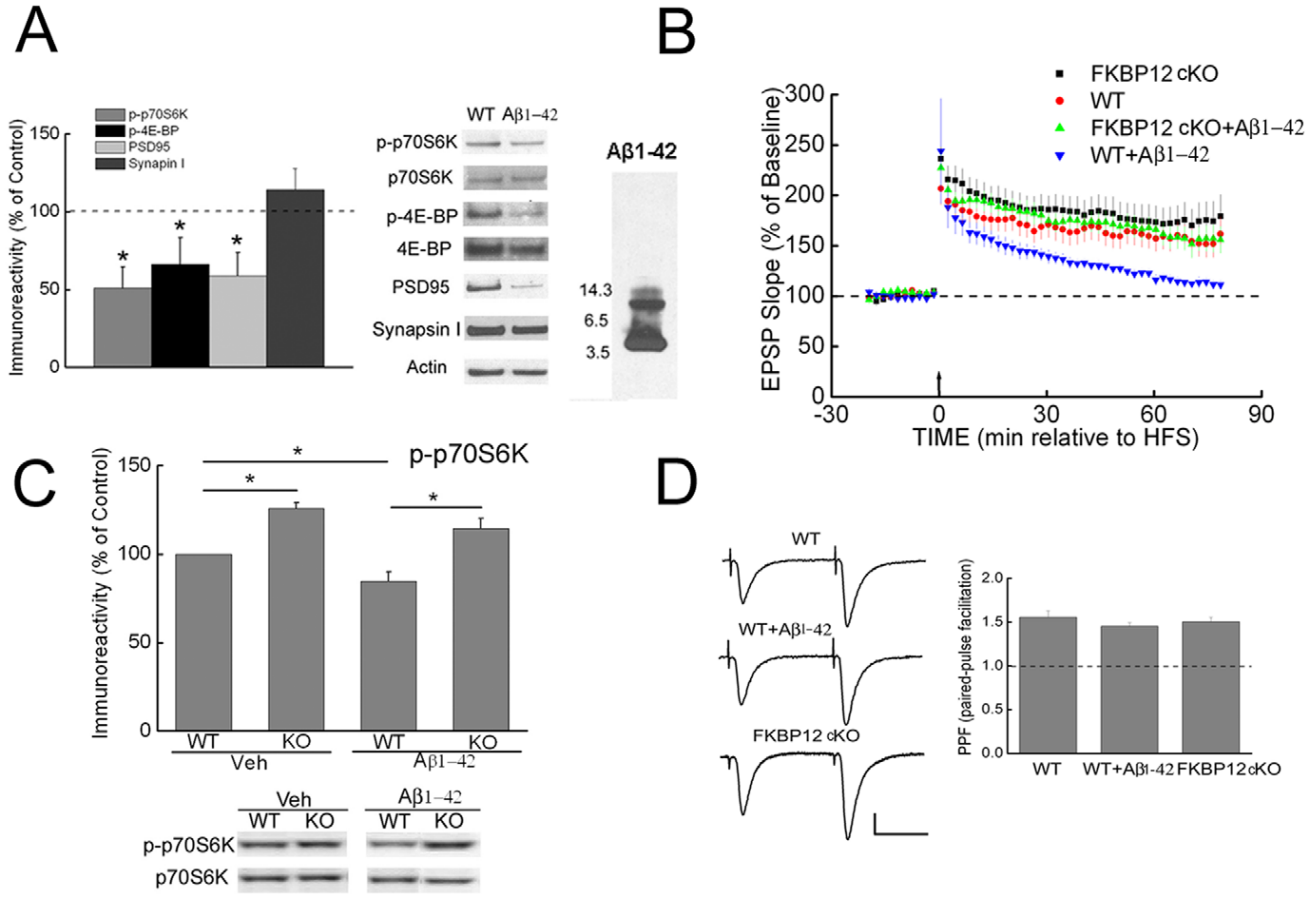
Inhibition of mTOR signaling and LTP by extracellular Aβ1-42 is prevented in FKBP12 cKO mice. (A) Treatment of slices from WT mice with exogenous Aβ1-42 (100 nM, 60 min) reduced levels of phospho-p70S6K, phospho-4E-BP, and PSD-95 compared to untreated WT slices (controls). n = 4. *p<0.05. Representative Western blot from three experiments after direct loading of the Aβ1-42 preparation (far right of the panel) showed mostly Aβ1-42 monomers, and also significant amounts of dimers and trimers. (B) LTP induced by HFS in WT mice (controls; red, n = 8) was blocked by treatment with Aβ1-42 (100 nM, blue, n = 7). In contrast, LTP was sustained in the presence of Aβ1-42 (100 nM, green) in FKBP cKO mice (n = 8). Aβ1-42 was present throughout the recording. (C) Inhibitory effects of Aβ1-42 on phospho-p70S6K were blunted in FKBP12 cKO mice. n = 5. *p<0.05. Slices were treated with Aβ1-42 for 60 min. (D) Slices from both FKBP12 cKO and WT mice treated with Aβ1-42 (60 min) exhibited normal PPF compared to the WT control. The percent facilitation, determined by the ratio of the second fEPSP to the first fEPSP (interpulse interval  = 50 ms), together with representative fEPSP traces are shown. n = 5 for WT; n = 6 for WT treated with Aβ; n = 8 for FKBP12 cKO. Scale bar, 0.5 mV/25 ms.

Numerous studies have shown that direct exogenous application of Aβ1-42 causes deficits in hippocampal synaptic plasticity, supporting a toxic role for soluble Aβ oligomers [1], [2]. Western blotting of our synthetic Aβ1-42 preparation indicated the presence of monomers and oligomers, predominantly dimers and trimers (Figure 4A), although we cannot exclude the possibility that during the incubation period higher molecular weight oligomer formation may have occurred. We previously reported that application of this Aβ1-42 preparation reduced levels of PSD-95 but not of synapsin I in cultured wild type neurons, analogous to the alterations in these proteins in Tg2576 compared to WT neurons in culture [20]. We now determined that levels of PSD-95 but not of synapsin I were also reduced in WT hippocampal slices by treatment with this preparation of synthetic Aβ1-42 (Figure 4A), paralleling the results seen in Tg2576 compared to WT hippocampal slices (Figure 1). Of note, treatment of WT hippocampal slices with this exogenous Aβ1-42 also caused a marked decrease in levels of phosphorylated p70S6K and 4E-BP (Figure 4A). Similar reductions in phosphorylated p70S6K and 4E-BP were seen in WT primary neurons treated with Aβ1-42 (data not shown).

To further examine mTOR signaling in Aβ-related synaptic dysfunction, we next utilized a mouse model in which the gene encoding FK506-binding protein 12 (FKBP12) is conditionally deleted in hippocampus and forebrain [29]. FKBP12 represses mTOR activity, and removal of FKBP12 was demonstrated to enhance mTOR signaling as well as LTP and memory [29]. For this set of experiments, we confirmed that treatment with exogenous synthetic Aβ1-42 caused inhibition of LTP in hippocampal slices of WT mice (Figure 4B). Consistent with prior data [29], slices from FKBP12 conditional KO (cKO) mice showed enhanced LTP compared to WT slices. Of note, and in contrast to slices of WT mice, when FKBP12 cKO slices were treated with Aβ1-42, LTP was now successfully elicited by HFS (Figure 4B), indicating that up-regulation of mTOR signaling from knocking out FKBP12 prevented Aβ-related impairment in synaptic plasticity. Supporting these electrophysiology data, Western blotting demonstrated that the decrease in levels of phosphorylated p70S6K seen in WT slices with Aβ1-42 treatment was prevented in slices from FKBP12 cKO mice (Figure 4C). PPF experiments confirmed that pre-synaptic function was not significantly altered in slices from FKBP12 cKO mice [29] or in slices from WT mice treated with Aβ1-42 (Figure 4D). Taken together, up-regulation of mTOR signaling via FKBP12 cKO prevented Aβ-related LTP impairment.

### Cellular co-localization of intraneuronal Aβ and components of the mTOR pathway

**Figure 5:**
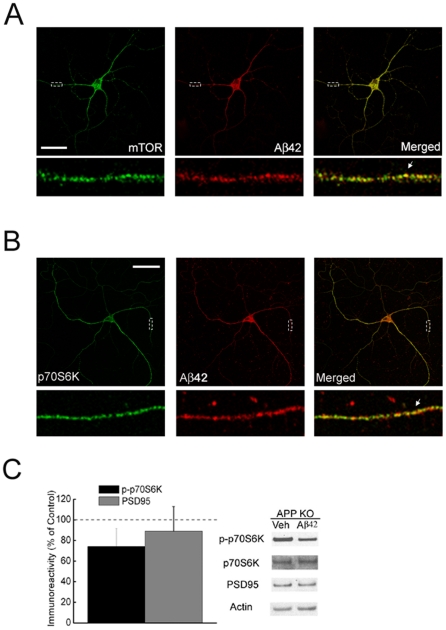
Cellular co-localization of intraneuronal Aβ and components of the mTOR pathway in Tg2576 neurons. (A and B) Immuno-fluorescence confocal microscopy of Aβ42 with either mTOR (A) or p70S6K (B) in Tg2576 neurons at 12 DIV. Note the punctate co-localization of intraneuronal Aβ42 with mTOR and p70S6K. Scale bar, 50 µm. (C) Aβ1-42 (100 nM, 60 min) treatment failed to induce inhibition of p70S6K phosphorylation and to reduce PSD-95 levels in slices from APP KO compared to WT (control) mice. n = 9. *p<0.05.

The mechanism(s) by which Aβ affects mTOR signaling in neurons from Tg2576 mice remain unclear. To investigate whether there might be a spatial relation between Aβ42 and mTOR, we performed confocal microscopy in cultured Tg2576 neurons. Previous studies have reported that both mTOR [30] and Aβ42 [31], [32] localize particularly to endosomes. Consistent with this, there was punctate co-localization of Aβ42 in neurites with both mTOR (Figure 5A) and p70S6K (Figure 5B). These findings place mTOR signaling components in the right place to be regulated by intraneuronal Aβ. To test whether this interaction might be a requirement for Aβ-induced mTOR impairment, we studied the effect of extracellular Aβ1-42 on mTOR in hippocampal slices from APP knockout mice [33]. We previously showed that synaptic toxicity of extracellular Aβ1-42 is blocked in APP knockout neurons, suggesting a requirement of intracellular APP processing and Aβ generation [19]. Remarkably, in hippocampal slices prepared from APP knockout mice, Aβ1-42 treatment failed to significantly alter levels of phosphorylated p70S6K or levels of PSD-95 (Figure 5C), supporting a role for APP processing and intraneuronal Aβ in down-regulation of mTOR signaling.

## Discussion

Impaired neuroplasticity due to abnormal accumulation of Aβ has been proposed as a key factor in the cognitive decline with AD pathogenesis, even preceding plaque formation or neurodegeneration [1], [2]. Developing a mechanistic understanding of the ability of soluble Aβ to interfere with synaptic plasticity, and in particular the stable, translation-dependent forms of plasticity that are thought to mediate LTM, could yield important insights into the pathophysiology of AD. mTOR-mediated protein synthesis is important for long-lasting forms of synaptic plasticity and memory [34]. In the present study, we demonstrate that mTOR signaling in the hippocampus is impaired in a transgenic mouse model of AD, an effect that is mimicked by treating hippocampi of normal mice with soluble Aβ1-42. Importantly, pharmacological or genetic up-regulation of mTOR signaling restored LTP in slices from Tg2576 mice and prevented Aβ-induced impairment in LTP. These findings provide novel insights into the mechanism of Aβ-related synaptic dysfunction underlying memory impairment in AD.

In agreement with the data with FKBP12 cKO mice, GSK3 inhibitors also rescued the Aβ-related LTP failure. Originally identified as a regulator of glycogen metabolism, GSK3 is a multifunctional serine/threonine kinase that has many potential substrates. It has been reported that GSK3 can inhibit mTOR through phosphorylation of TSC2 [27]. Furthermore, GSK3 is highly expressed in hippocampus and is implicated in synaptic plasticity [35], [36]. In fact, GSK3 dysfunction has been proposed as an important underlying molecular mechanism for AD pathogenesis and accordingly has stimulated interest in the development of therapeutically useful GSK3 antagonists [35], [37]–[39]. In our study, the rescue of LTP by GSK3 inhibitors was sensitive to rapamycin, consistent with GSK3 acting to regulate mTOR signaling.

Inhibition of mTOR signaling was reported in the brains of another AD transgenic mouse model [12]. On the other hand, mTOR signaling was reported to be up-regulated in postmortem human AD brains, particularly in tangle bearing neurons [21]. We confirmed isolated phospho-p70S6K increases in AD vulnerable neurons of human AD brain, which was particularly prominent in tangle-bearing neurons (Supplementary Figure S3). Of note, we observed reductions in phospho-p70S6K only at early but not at later ages in brains of Tg2576 mice. It is possible that up-regulation of mTOR noted in human AD [12] and 3xAD-Tg mice [13] develops later secondary to another process, such as tau alterations, cell cycle re-entry and/or inflammation; for example, the latter is known to stimulate mTOR signaling [40]. In addition, although it is well established that acute inhibition of mTOR impairs synaptic plasticity, it was recently reported that chronic treatment with rapamycin improved behavior of AD transgenic mice [13], [14], although effects were mild. Acute compared to chronic treatment may be critical in explaining the differences among studies. Of note, there are two mTOR complexes, mTOR complex 1 (mTORC1) and complex 2 (mTORC2). The mTORC1 complex plays a critical role in synaptic plasticity. In contrast, nothing is currently known about the role of mTORC2 in synaptic plasticity [7]. Prolonged but not acute treatment with rapamycin has been reported to lead to interference with mTORC2 [41], which has functions that are independent of mTORC1 [42]. In light of these studies, we hypothesize that there is a complex relationship between aging, Aβ, and dysregulation of the mTOR pathway in AD.

Of note, there are many other examples of similarly contradictory-appearing data with respect to signaling in other common age-related diseases. For example, a study reporting down-regulation of mTOR signaling in huntingtin-accumulating neurons in a mouse model of Huntington's disease at the same time showed that treating the mice with rapamycin was protective [43]. In another age-related disease, adult onset diabetes, defective insulin signaling through the insulin-PI3K-Akt pathway, which is upstream of mTOR, is well-established, although inhibition of this same pathway is known to extend lifespan in yeast and C. elegans models [44]. Of note, similar conflicting data also exist on the insulin-PI3K-Akt pathway in AD models. On one hand, Aβ was shown to impair insulin-PI3K-AKT signaling [2], [45]–[47] and insulin treatment was reported to improve cognitive function in patients with early AD [48], while on the other hand, AD transgenic mice with reduced insulin signaling were reported to be protected against cognitive decline [49].

Although the precise molecular mechanism whereby Aβ alters the mTOR pathway is not known, our finding that Aβ42 co-localizes with mTOR and p70S6K within neurites of APP mutant transgenic mice places it in the right location to dysregulate mTOR signaling. Recent evidence supports that extracellular Aβ acts via APP processing and potentially also intraneuronal Aβ42 [19], and therefore we examined whether extracellular Aβ1-42 down-regulated mTOR in APP knockout neurons. Remarkably, the effect of exogenous Aβ on mTOR signaling was blocked in APP knockout neurons, further supporting an emerging complex link between extracellular and intracellular Aβ.

Given the pivotal role of mTOR in maintaining cellular and organismal homeostasis by controlling multiple fundamental biological processes, perturbing the balance in mTOR signaling might be key to better understanding the Aβ-related impairment in signaling involved in the synaptic dysfunction that characterizes AD.

## Materials and Methods

### Hippocampal slice preparation and electrophysiology

All experiments were conducted according to and approved by the Weill Cornell Medical College IACUC (Protocol# 0610-550A). For preparation of acute hippocampal slices, 3 to 4 month-old Tg2576 or wild type mice were first deeply anesthetized with isoflurane and then decapitated. Brains were rapidly removed and placed in ice-cold artificial cerebrospinal fluid (ACSF) containing (in mM) 118 NaCl, 3.5 KCl, 2.5 CaCl2, 1.3 MgSO4, 1.25 NaH2PO4, 24 NaHCO3, and 15 glucose, bubbled with 95% O2/5% CO2. The hippocampus was then quickly dissected out and 400 µm thick transverse slices were made on a tissue chopper at 4°C. The slices were maintained in an interface chamber (ACSF and humidified 95% O2/5% CO2 atmosphere) at room temperature for at least 2 hours before removal for experiments. For electrophysiological recording, slices were transferred to a submersion chamber preheated to 30–32°C, where they were superfused on a nylon mesh with ACSF. Monophasic, constant-current stimuli (100 µsec) were delivered with a bipolar stainless steel electrode placed in the stratum radiatum of the CA3 region, and the field excitatory postsynaptic potentials (fEPSPs) were recorded in the stratum radiatum of the CA1 region with electrodes filled with ACSF (Re = 2–4 MΩ). The fEPSPs were monitored by delivering stimuli at 0.033 Hz, and the signal was low-pass filtered at 3 kHz and digitized at 20 kHz. fEPSPs were acquired, and amplitudes and maximum initial slopes measured, using either Axobasic routine or pClamp 9 (Axon Instruments, Foster City, CA). High Frequency Stimulation (HFS) protocol consisting of two 1-s long 100 Hz trains, separated by 20 s, delivered at an intensity that evoked a 1.5 mV fEPSP, was used to induce LTP. In some experiments, “weak” HFS was used, consisting of a single 1 s long 100 Hz train delivered at an intensity that evoked a baseline fEPSP between 0.5 and 0.6 mV.

### Primary neuronal cultures

Primary neuronal cultures were prepared from cortices and hippocampi of embryonic day (E) 16 Tg2576 and wild-type littermate mice as described [19]. One pup corresponds to one set of cultures, and genotyping was done by PCR of the cerebellum. Primary neurons were used at 12 days in vitro (DIV).

### Drug treatments

Drugs were prepared as stock solutions and diluted to final concentrations before use. For hippocampal slices, drug incubation was performed at 30–32°C in submersion maintenance chambers containing ACSF saturated with bubbling 95% O2/5% CO2. The final concentrations and sources were as follows: forskolin (50 µM, Sigma), DHPG (50 µM, Sigma), Rapamycin (1 µM, Calbiochem), kenpaullone (1 µM, Calbiochem), and LiCl (10 mM, Sigma). Aβ1-42 stock (100 µM, Tocris) was prepared in water and stored at −20°C overnight before use at a final concentration of 100 nM.

### Western blotting

Lysates were prepared from either primary neurons at 12 DIV or hippocampal slices. Previously described protocols were followed [19], [20]. The following inhibitors were added into the lysis buffer (in mM, unless indicated otherwise): 25 Na fluoride, 2 Na pyrophosphate, phosphatase inhibitor cocktail I & II (Calbiochem), protease inhibitor cocktail (Roche). Protein concentrations were determined by the Bradford technique (Bio-Rad Laboratories), and equal amounts of protein from each sample were loaded on 4–12% Tris-Glycine SDS-PAGE (Invitrogen) gels. After transfer, membranes were blocked for at least 30 minutes at room temperature with blocking buffer [BB; 5% non fat dry milk in TBS containing 0.1% Tween 20 (TBS-T)], then probed overnight at 4°C using primary antibodies for phospho(Thr389)-p70S6K (1∶1000; Cell Signaling), p70S6K (1∶1000; Cell Signaling), phospho(Thr37/46)-4E-BP (1∶1000; Cell Signaling), 4E-BP (1∶1000; Cell Signaling), PSD-95 (1∶1000, Chemicon), Synapsin I (1∶2000, Upstate), or actin (1∶10000; Sigma-Aldrich). Densitometric analysis of the bands was performed using Scion Image Software. Data were analyzed using *student* t-test with Origin (OriginLab Corp.) software, with significance placed at *p*<0.05. Measurements of phospho-proteins were normalized to corresponding total proteins. Summary data were presented as group means with standard error bars.

### Immunofluorescence confocal microscopy

Ice-cold 4% paraformaldehyde was used to fix both primary neurons (20 min) and slices (overnight). The slices were further cut into 30 µm sections. Protocols were followed as described [19], [20]. Fixed neurons, hippocampal slice sections or postmortem human brain sections were incubated overnight at 4°C with the following antibodies: human specific Aβ42 (1∶250; Signet), phospho(Thr389)-p70S6K (1∶250; Cell Signaling), p70S6K (1∶250; Cell Signaling), and mTOR (1∶100; Cell Signaling).

## Supporting Information

Figure S1 (A) HFS induced normal LTP in slices from 2-month-old Tg2576 mice, comparing to WT mice. n = 4. (B) Western blotting showed no reduction of phospho-p70S6K and PSD95 in slice from 2-month-old Tg2576 mice. n = 4.
(2.73 MB TIF)

Figure S2 (A) Weak HFS (one train) induced similar early LTP in slices from 3–4-month-old Tg2576 mice and WT mice that decayed to baseline in about 80 minutes. n = 5. (B) Slices from 3–4-month-old Tg2576 mice demonstrated normal PPF. n = 5. Scale bar, 0.5 mV/25 ms.
(2.56 MB TIF)

Figure S3 Increased labeling of phospho-p70SK6 in AD vulnerable neurons of the hippocampus in a case with AD (A) compared to a control (B). Inserts represent higher power views of the black boxes within the lower power images. The inset in (A) reveals tangle-like labeling of phospho-p70SK6 in CA1 pyramidal neurons. Scale bar, 1 mm.
(8.75 MB TIF)
