# Supplementary figures and images for: Dysregulation of the mTOR Pathway Mediates Impairment of Synaptic Plasticity in a Mouse Model of Alzheimer's Disease
Source: PLoS One. 2010 Sep 20;5(9):e12845. doi: 10.1371/journal.pone.0012845 (PMC2942840; doi:10.1371/journal.pone.0012845)

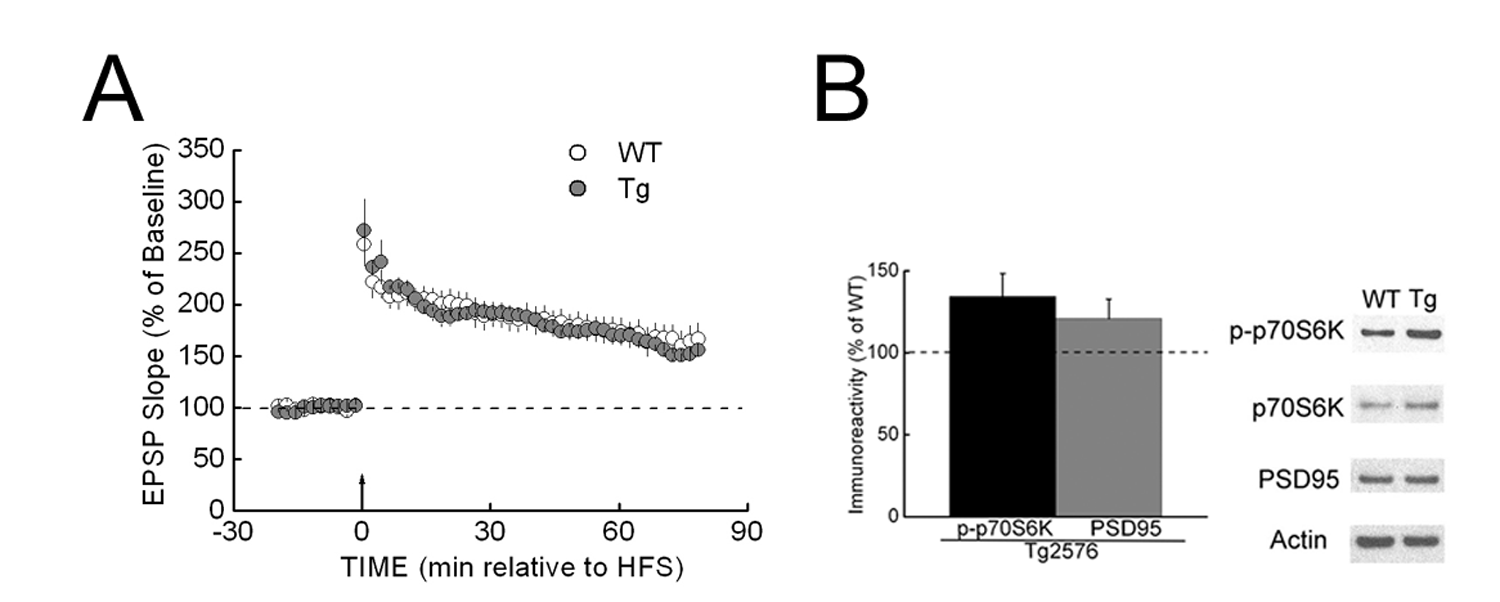

Supplement: Figure S1 — (A) HFS induced normal LTP in slices from 2-month-old Tg2576 mice, comparing to WT mice. n = 4. (B) Western blotting showed no reduction of phospho-p70S6K and PSD95 in slice from 2-month-old Tg2576 mice. n = 4. (2.73 MB TIF) [file pone.0012845.s001.tif]

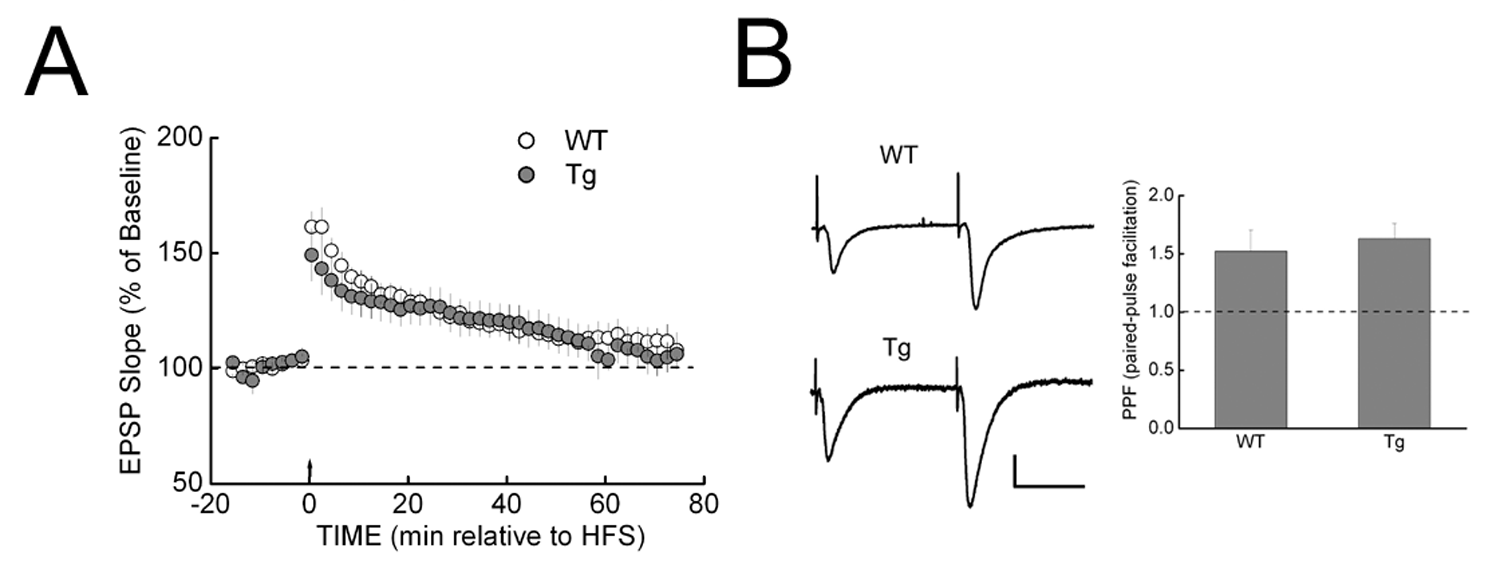

Supplement: Figure S2 — (A) Weak HFS (one train) induced similar early LTP in slices from 3–4-month-old Tg2576 mice and WT mice that decayed to baseline in about 80 minutes. n = 5. (B) Slices from 3–4-month-old Tg2576 mice demonstrated normal PPF. n = 5. Scale bar, 0.5 mV/25 ms. (2.56 MB TIF) [file pone.0012845.s002.tif]

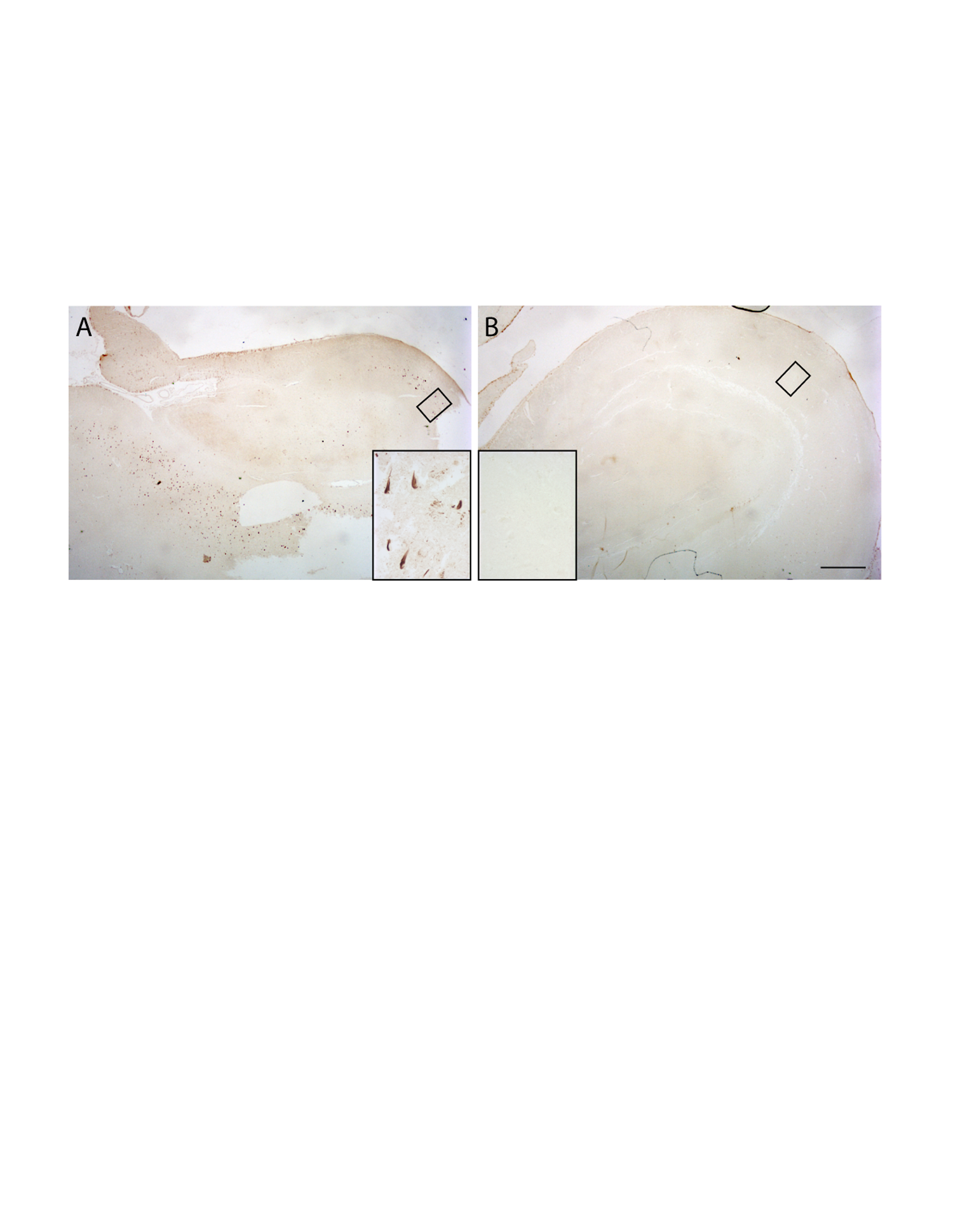

Supplement: Figure S3 — Increased labeling of phospho-p70SK6 in AD vulnerable neurons of the hippocampus in a case with AD (A) compared to a control (B). Inserts represent higher power views of the black boxes within the lower power images. The inset in (A) reveals tangle-like labeling of phospho-p70SK6 in CA1 pyramidal neurons. Scale bar, 1 mm. (8.75 MB TIF) [file pone.0012845.s003.tif]
